# Vitamin B and Zinc Supplements and Capsaicin Oral Rinse Treatment Options for Burning Mouth Syndrome

**DOI:** 10.3390/medicina57040391

**Published:** 2021-04-17

**Authors:** Viktors Jankovskis, Guntars Selga

**Affiliations:** RSU Institute of Stomatology, Department of Oral Medicine, Rīga Stradiņš University, LV-1007 Riga, Latvia; selga@sveiks.lv

**Keywords:** glossodynia, burning mouth syndrome, vitamin B supplements, zinc supplements, capsaicin

## Abstract

*Background and Objectives*: Burning mouth syndrome (BMS) is an enigmatic, idiopathic, chronic, often painful clinical entity, where patients experience oral burning without clear clinical changes on the oral mucosa. There are yet to be well established standardized and validated definitions, diagnostic criteria or classifications for burning mouth syndrome. The aim of this study is to determine whether vitamin B complex and zinc supplements or 0.02% topical capsaicin rinse, can help alleviate BMS pain/burning levels. The objectives: (1) Gather data before and after treatment with vitamin B complex and zinc supplements (2) After the vitamin B complex and zinc protocol gather data before and after treatment with 0.02% topical capsaicin rinse (3) Data analysis and assessment for both treatment methods. *Materials and Methods*: 89 patients took part in the vitamin B and zinc supplement treatment regimen, out of those 20 patients took part in the capsaicin rinse treatment regimen. Before and after each treatment pain/burning levels were determined using the Visual analogue scale, salivary flow was also determined. *Results*: Both treatment methods showed statistically significant data in reducing pain/burning levels. There were no statistically significant changes in the salivary flow after any treatment. *Conclusion*: vitamin B and zinc supplement therapy and topical capsaicin rinse therapy can be an effective way to decrease pain/burning sensation levels in patients with BMS. More research should be conducted to determine the benefit of either vitamin B and zinc supplement therapy or topical capsaicin rinse therapy, so that BMS patients have treatment options, that have as few side-effects as possible.

## 1. Introduction

Burning mouth syndrome (BMS) is an enigmatic, idiopathic, chronic, often painful clinical entity, where patients experience oral burning without clear clinical changes on the oral mucosa. There are yet to be well established standardized and validated definitions, diagnostic criteria or classifications for burning mouth syndrome [1,2]. Burning sensation mostly affects the tongue, but can also affect the hard palate, the lower and upper lip [2,3,4]. A patient may experience a symptom triage—burning sensation, dysgeusia and xerostomia, but for a patient to experience all three of the symptoms at the same time is a rarity [5]. BMS is more common among women [4,6]. Prevalence rates increase after the age of 50 [4,7,8]. The prevalence of BMS in the population is 0.7–15%, depending on the diagnostic criteria used in research [2]. The incidence rate of BMS, determined for 11 years from 2000 through 2010 was 11.4 per 100,000 person-years [4].

At present there are 3 approaches for the treatment of BMS: behavioral strategies, topical therapies (anxiolytics–clonazepam; analgesics–capsaicin; anesthetics–lidocaine), systemic therapies (antidepressants–paroxetine; anxiolytics–clonazepam, diazepam; anticonvulsants–gabapentin, pregabalin; salivary stimulants–pilocarpine; vitamin B, C supplements). Despite having randomized clinical trials reviewing management approaches and providing evidence-based treatment algorithms, the results to treat BMS remain inconclusive [2].

Research has reported that patients with BMS may have decreased levels of vitamins B1 (thiamine), B2 (riboflavin), B6 (pyridoxine), B12 (cobalamin) [9,10,11]. A decrease of B1, B6, B12 can be considered as a cause of neurological disorders such as neuropathy [2,12]. Decreased zinc levels have also been reported among patients diagnosed with BMS [10,13,14,15]. Vitamin B and zinc supplementations have been mentioned as a treatment options for BMS patients [2].

Recent research has reported that topical capsaicin was used as an analgesic for the treatment of BMS with positive results [16,17,18]. BMS appears to be associated with increased levels of nerve growth factor (NGF) in the nerve fibers and increased expression of cation channel vanilloid subfamily member 1 (TRPV1) (also known as the capsaicin receptor) fibers in the tongue papillae [19]. Capsaicin provides analgesic and anti-inflammatory effects on sensory neurons, and increases saliva production and provides anti-inflammatory properties in the salivary glands. Capsaicin has also displayed antitumoral effects in gastrointestinal cancers [20,21].

The aim of this study is to determine, whether vitamin B complex and zinc supplements or 0.02% topical capsaicin rinse, can help alleviate BMS pain/burning levels.

## 2. Materials and Methods

The research data was acquired between 31 July 2018 and 22 September 2020. The clinical trial was conducted at the Institute of Stomatology (Stomatoloģijas institūts), Riga, Latvia. This controlled clinical trial is part of the doctoral thesis, which has been approved by the Research Ethics Committee of Rīga Stradiņš University (RSU). The research article has been made in accordance with the Helsinki declaration. All subjects gave their informed consent for inclusion before participation in the study. Overall 89 patients (8 males and 81 female) took part in the vitamin B and zinc supplement treatment regimen, out of those 20 patients took part in the capsaicin rinse treatment regimen.

### 2.1. Inclusion Criteria:

Any type of oropharyngeal symptom that can be persistent or intermittent with possible phases of remission/exacerbation during the day

- Absence of any clinically and instrumentally detectable oropharyngeal lesion
- Absence of any type of local and/or systemic factor, such as oral diseases, drugs, trauma, hypersensitivity reactions, physical/chemical agents
- In addition (but not mandatory): symptoms are persistent (typically 3 months) [22].

### 2.2. Assessment Method:

To determine the pain/burning sensation intensity, the visual analogue scale (VAS) (scale of 0–10) was used [23]. During appointments patients were required to mark pain/burning levels for the morning, afternoon and evening periods before any treatment was started and after either of the treatment procedures were finished.

Salivary flow was determined during the appointments with a doctor before and after treatment procedures. Patients were required to collect saliva into 2 tubes (one for unstimulated saliva and one for stimulated saliva) for 5 min each. The flow of the unstimulated saliva was observed when the patient was at rest. The stimulated saliva flow was collected after the patient was chewing a piece of paraffin to stimulate salivation. The norm was set at 0.2 mL/min for unstimulated saliva flow and 0.5–2 mL/min for stimulated saliva flow. If patients had an unstimulated salivary flow of <0.2 mL/min and stimulated flow < 0.5 mL/min, then it was confirmed that the patient had hyposalivation [24].

Additionally, these parameters were also determined—the median age and sex of the patients, the most affected area, median duration of the disease, number of patients with a 50% recovery and full recovery.

### 2.3. Treatment Method Protocols

#### 2.3.1. Vitamin B and Zinc Supplement Therapy Protocol

During the study patients received two supplements at the same time:

(a) Zinc supplement pill (15 mg Zinc, 35 mg Vitamin C, Glucose, Fructose, Magnesium Stearate (E470b)). 1 pill—500 mg. Patients ingested 1 pill a day for 1 month in the morning.
(b) Vitamin B complex supplement pill (35 mg thiamine (vitamin B1), 35 mg riboflavin (vitamin B2), 35 mg niacin (vitamin B3), 35 mg Pantothenic acid (vitamin B5), 25 mg pyridoxine (vitamin B6), 10 μg vitamin B12, 600 μg Folic acid (vitamin B9), 150 μg Biotin (vitamin B7). 1 pill—370 mg. Patients ingested 2 pills a day for 1 month in the morning and in the evening.

In this study the dosages of said supplements did not exceed the recommended dosages as presented in the text “Tolerable Upper Intake Levels for Vitamins and Minerals” done by the European food and safety authority (EFSA). A combined treatment was chosen, so that patients did not have to wait even longer to receive Capsaicin treatment, in the case if the treatment with either zinc or Vitamin B supplements would not be sufficient. Patients only receiving only one treatment option would lead to a higher patient dissatisfaction and a risk of leaving the study, without receiving any other possible treatment options. Pain/burning levels were determined before and after treatment in all three-time frames (morning, afternoon, evening). Salivary flow levels were determined before and after treatment during the appointment.

#### 2.3.2. Capsaicin Rinse Therapy Protocol

If the patients’ treatment was not effective after the Vitamin B and Zinc Supplement Therapy Protocol, patients received a treatment regimen with a 0.02% capsaicin rinse [16]. The patients were required to rinse the mouth 3 times a day for 30 s after meals. The treatment regimen was 3 weeks, after which patients had their pain/burning levels, and salivary flow levels evaluated repeatedly in the same manner as after the Vitamin B and Zinc Supplement Therapy Protocol.

### 2.4. Statistical Analysis

Data was analyzed using the Statistical Package for Social Sciences (SPSS for Windows, version 23.0, SPSS Inc., Chicago, IL, USA). For nonparametric data, the Wilcoxon test was used for variable comparison before and after each treatment method. The adopted significance level was 5% (*p* < 0.05). Overall patient data and data frequencies were also checked. To check correlation, the Spearman correlation test was used.

## 3. Results

### 3.1. Overall Patient Data

#### 3.1.2. Age

Median age for 89 of the patients in the study was 59 years (interquartile range (IQR) 19).

#### 3.1.3. Affected Areas

The most affected area by the burning sensation was the tip of the tongue, affecting 77 patients (86.5%, Confidence interval (CI) 77.6–92.8%), followed by the sides of the tongue, affecting 56 patients (62.9%, CI 52.0–72.9%), ventral surface of the tongue, affecting 30 patients (33.7%, CI 24.0–44.5%), lips, affecting 24 patients (26.9%, CI 18.1–37.4%), palate, affecting 19 patients (21.3%, CI 13.3–32.3%) and cheeks, affecting 7 patients (7.9%, CI 3.3–15.2%).

#### 3.1.4. Duration of Disease

The median duration of disease was 6 months (IQR 9).

### 3.2. Pain/Burning Levels Before and After Vitamin B and Zinc Supplement Therapy

#### 3.2.1. Morning

Median pain/burning sensation levels in the morning before the treatment had a score of 2 (IQR 3). After the treatment the score lowered to 1 (IQR 2). 52 patients (58.43%, CI 47.5–68.6%) noted improvement, 6 (6.7%, CI 2.5–14.5%) noted worsening, 31 (34.8%, CI 25.0–45.6%) had no change in pain/burning sensation levels, Z = −5.503, *p* < 0.000.

#### 3.2.2. Afternoon

Median pain/burning sensation levels in the afternoon before the treatment had a score of 4 (IQR 2). After the treatment the score lowered to 2 (IQR 2.5). 61 patient (68.54%, CI 57.8–77.9%) noted improvement, 5 (5.62%, CI 1.8–12.6%) noted worsening, 23 (25.8%, CI 17.1–36.2%) noted no change, Z = −6.156, *p* < 0.000.

#### 3.2.3. Evening

Median pain/burning sensation levels in the evening before the treatment had a score of 5.5 (IQR 3). The level score lowered to 3 (IQR 3) after the treatment. 60 patients (67.48%, CI 56.5–76.9%) noted improvement, 6 (6.7%, CI 2.5–14.5%) noted worsening, 23 (25.8%, CI 17.1–36.2%) noted no change, Z = −6.259, *p* < 0.000.

#### 3.2.4. Overall Pain Data

**Figure 1:**
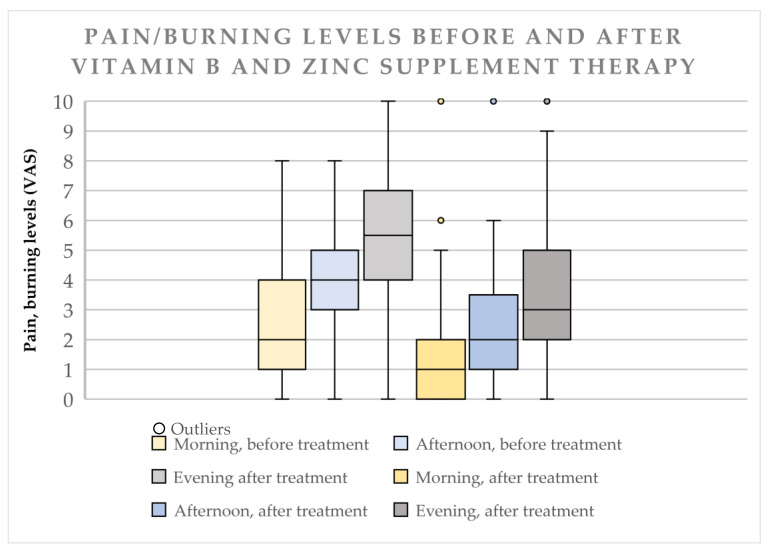
Pain/Burning Levels Before and After Vitamin B and Zinc Supplement Therapy. The dots represent “outliers”. It shows that specific patient findings were beyond the Interquratile range.

Data shows an increase of pain/burning sensation throughout the day, from morning till evening with a score rising from 2 to 5.5 before the treatment and 1 to 3 after the treatment, but overall data shows lowered pain/burning sensation levels for 58.43% of the patients in all 3 time periods after receiving the treatment. Table 1 and Figure 1.

### 3.3. Pain/Burning Levels Before and After Oral Capsaicin Rinse Therapy

#### 3.3.1. Morning

The median pain/burning sensation score in the morning period for the 20 patients after the vitamin B and zinc supplement therapy protocol was 2 (IQR 1). After the treatment with topical capsaicin the median lowered to 0 (IQR 2). 9 patients (45%, CI 27.2–72.8%) noted improvement, 1 (5%, CI 0–24.8%) noted worsening, 10 (50.0%, CI 27.2–72.8%) had no change in pain/burning sensation levels, Z = −1.996, *p* = 0.046.

#### 3.3.2. Afternoon

In the afternoon period the median pain/burning sensation score for the 20 patients after vitamin B and zinc supplement therapy protocol was 3 (IQR 1). After the treatment with capsaicin the median was 1.5 (IQR 3). 10 patients (50% CI 27.2–72.8%) noted improvement, 2 (10%, CI 1.2–31,7%) noted worsening, 8 (40%, CI 19.9–63.9%) had no change in pain/burning sensation levels, Z = −2.613, *p* = 0.009

#### 3.3.3. Evening

In the evening period the median pain/burning sensation score for the 20 patients after vitamin B and zinc supplement therapy protocol was 4 (IQR 3). After the treatment with capsaicin the median was 2 (IQR 6). 10 patients (50% CI 27.2–72.8%) noted improvement, 2 (10%, CI 1.3–31.7%) noted worsening, 8 (40.0%, CI 19.9–63.9%) had no change in pain/burning sensation levels, Z = −2.789, *p* = 0.005.

#### 3.3.4. Overall Pain Data

**Figure 2:**
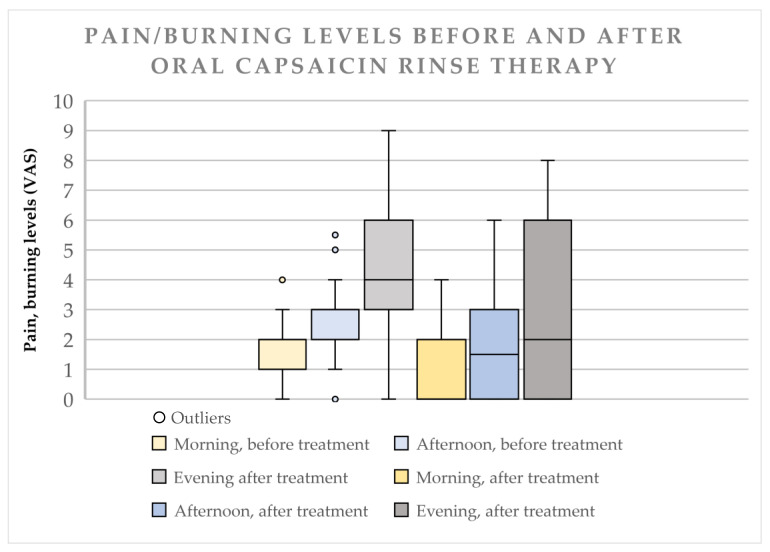
Pain/Burning Levels Before and After Oral Capsaicin Rinse Therapy. The dots represent “outliers”. It shows that specific patient findings were beyond the Interquratile range.

Data shows an increase in pain/burning sensation throughout the day, from morning till evening with a score rising from 2 to 4 before the treatment and 0 to 2 after the treatment, but overall data shows lowered pain/burning sensation levels for 9 (45.0%, CI 23.0–68.4%) patients in all three time periods of the day after receiving the treatment. Table 2 and Figure 2.

### 3.4. Salivary Flow Levels Before and After Treatment

Before the treatment procedures we assessed salivary flow rates for 48 (53.9%, CI 43.0–64.5%) of our patients, that complained of reduced salivary flow. Their salivary flow rate for unstimulated saliva was 0.2 mL/min (IQR 0.19) and for stimulated saliva—0.8 mL/min (IQR 0.64), showing that patients had a normal within range unstimulated and stimulated salivary flow rate. The salivary flow was determined a second time after vitamin B and zinc treatment regimen, where the unstimulated salivary flow rate stayed at 0.21 mL/min (IQR 0.28) and stimulated salivary flow rate increased to 1 mL/min (IQR 0.53). 28 patients (58.33%, z = −2.908, *p* = 0.04) showed an improvement of unstimulated salivary flow and 31 patient (64.58%, z = −3.705, *p* = 0.000) showed an improvement of stimulated salivary flow, the rest of the patients noted either no changes or worsening.

In the capsaicin rinse treatment regimen group the salivary flow levels were checked for 10 patients, where the lowered unstimulated salivary flow increased from 0.14 (IQR 0.21) ml/min (before the treatment) to 0.225 mL/min (IQR 0.41) (after treatment), with 7 patients (70%) noting improvement (z = −1.955, *p* = 0.051). The stimulated salivary flow median levels went from 0.94 (IQR 0.85) to 0.91 mL/min (IQR 0.61), with 7 patients (70%) noting improvement z = −1.486, *p* = 0.137).

**Figure 3:**
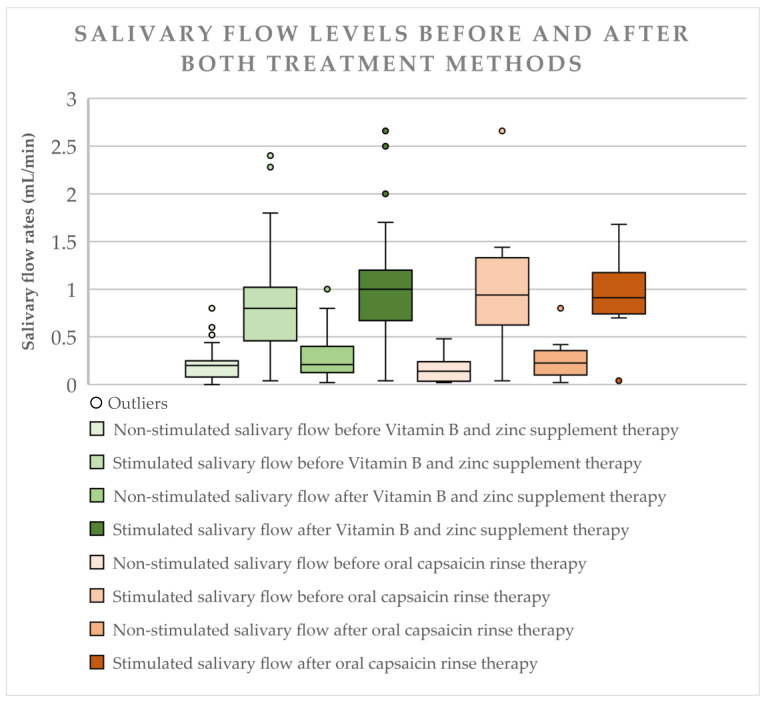
Salivary Flow Levels Before and After Treatment. The dots represent “outliers”. It shows that specific patient findings were beyond the Interquratile range.

Data shows changes in salivary flow for non-stimulated and stimulated saliva. Table 3 and Figure 3.

## 4. Discussion

This study shows that the most affected area by the burning/pain sensation was the tongue, particularly the tip, followed by the lips and palate. Other studies showed a similar pattern—most commonly affected was the tongue (affecting more than 50%), followed by the mucosal surface of the lower lip and anterior hard palate [3,4], or the tongue as being the most affected area, then the buccal mucosa, then the hard palate [7].

In this study the median age of patients was 59 (IQR 19) years (mean 57.66 ± Standard deviation (SD) 13.161), the female/male ratio was 10:1. Similarly another study showed, that the mean age of patients with BMS was 61.17 (SD ± 11.75) and the female/male ratio = 3:1 [6]. The data compared between this study and our study shows, that the data is similar in regards that BMS mostly affects patients over 55. Another study reported that the majority of the 169 patients were female (84.0%), with a female/male ratio of 5.2:1 [4]. Comparing between our study and literature, it shows that females more commonly seek help in regard to BMS, although in this study the ratio was even larger. In our study the median duration of the disease was 6 (IQR 9) months.

In literature the mean severity of BMS pain has been assessed at about 5–8 cm (or 50–80 mm) on a 10 cm (100 mm) Visual Analogue Scale (VAS), with mean VAS pain levels of 7.5 in women and 6.11 in men [5,7]. In our study the mean before any treatment method varied from a median of 2–5.5 points (mean 2.494 (SD ± 1.9136)—5.208 (SD ± 2.2256)) depending on the time frame of the day. Comparatively in our study people arrived to receive treatment with lower scored pain/burning levels compared to other data.

Before the treatments, the median levels of pain/burning sensation increased throughout the day. After the vitamin B and zinc supplement therapy there was a decrease in burning sensation in all three-time frames of the day, but still showing that the pain/burning sensation increased throughout the day, with it being the lowest in the morning and highest in the evening. For those patients without or inadequate improvement after the Vitamin B and Zinc Supplement therapy, oral capsaicin therapy was implemented.

After oral capsaicin rinse therapy there was a decrease of pain/burning sensation, but still presented an increase of pain/burning sensation from the morning period till the evening.

The vitamin B and zinc supplement therapy showed a decrease by 1 point, 2 points, 2.5 points of pain/burning levels for the morning, afternoon, evening respectively, but the oral capsaicin rinse therapy showed decrease by 2 points, 1.5 points, 2 points of pain/burning levels for the morning, afternoon, evening respectively, overall showing, that both treatment regimens had differences in their ability to change the scores of VAS for pain/burning sensation.

A larger percent of patients fully recovered using the topical capsaicin protocol (20%) compared to vitamin B and zinc treatment regimen (7.8%), although there were less patients in that specific group. Both treatment options showed results in alleviating pain for patients.

Those patients, that did not take part in the oral Capsaicin rinse therapy after receiving Vitamin B and zinc supplements, showed a decrease of pain/burning levels by 1 point for the morning period, by 2 points during the afternoon, by 2 points during the evening. Those patients that after vitamin B and zinc treatment regimen did take part in the oral Capsaicin rinse therapy, after the Vitamin B and zinc treatment regimen showed no changes of pain/burning levels for the morning period, decrease by 1 point during the afternoon, by 2 during the evening (Table A1 in Appendix A).

The benefits of vitamin supplements in neuropathic pain treatment can be divided in two aspects: those decreasing damage effects on the nervous fiber (vitamin B12) and those that are antinociceptive and antihyperalgesic (vitamin B1, B6) [12]. It has been previously reported that patients with BMS, who had a deficiency of vitamins B1, B2 and B6 received either vitamin B supplements or a placebo therapy, but no effect on BMS of vitamin replacement therapy or placebo therapy could be demonstrated [25]. Lamey [9] reported a deficiency in vitamins B1, B2, and/or B6 in some BMS patients with encouraging therapeutic results following vitamin replacement therapy. However, a proper control group for assessment of a possible placebo effect was missing in that study. Another study reported that B12 oral replacement (1 mg to 2 mg per day) was as effective as parenteral administration for correcting anemia and neurologic symptoms [26]. Additionally, Scala [5] cited two older research papers that describe vitamin B complex replacement therapy, however often proving an ineffectiveness for pain relief (Hugoson and Thorstensson, 1991; Dutree-Meulenberg et al., 1992).

The essential trace element zinc (Zn) has many physiologic roles, being required for growth and functioning of the immune system [27]. It can be postulated that zinc deficiency and lower serum zinc level are involved in the pathogenesis of oral mucosal diseases [13]. Cho [14] in an animal study reported that zinc deficiency can be a potential causal factor for BMS, however it has not been found to play a definitive role in the etiology of BMS. A study reported that Zinc-replacement therapy was effective in reducing pain at 6 months in patients with BMS and zinc deficiency [28]. Cho [14] reported that zinc replacement therapy had a greater effect than other symptomatic treatments in patients with zinc deficiency.

Capsaicin is known to act on the transient receptor potential cation channel vanilloid subfamily member 1 (TRPV1), which is involved in somatic and visceral peripheral inflammation, in the modulation of nociceptive inputs to spinal cord and brain stem centers, as well as the integration of diverse painful stimuli. Topical capsaicin medications are used for the treatment of painful states derived from neuralgia, diabetic neuropathy, osteoarthritis and rheumatoid arthritis providing analgesic and anti-inflamatory effects on sensory neurons. It has also been reported, that capsaicin can result in an increase of salivary flow by affecting TRPV1 receptors [20,29]. Research has also shown potential that capsaicin has the ability to reduce tumor cell proliferation and promotes their apopotosis in cases of gastrointestinal cancers [21]. BMS appears to be associated with increased levels of nerve growth factor in nerve fibers and expression of TRPV1 [19].

In two studies, where a capsaicin oral rinse and an oral capsaicin gel was used, patients experienced pain relief [16,17]. Although capsaicin can help in relieving pain effect for the short term, studies have noted, that it did not provide a complete remission of symptoms for some of the patients. The patients would return within a certain time frame [16,17]. One study noted using systemic capsaicin to reduce burning mouth symptoms for patients, but only effective for a short term [18]. Topical capsaicin is unlikely to help substantially, as some of the symptoms are likely to arise from deeper fibers [19]

In this study it was observed that patients with BMS have a normal non-stimulated salivary flow and stimulated salivary flow and it was also observed that there was a small improvement of non-stimulated and stimulated salivary flow after vitamin B and zinc supplement therapy. Patients who took part in the oral capsaicin rinse therapy had a small increase from lowered to normal levels of non-stimulated salivary flow and a decrease of stimulated salivary flow (within the range of normal salivary flow). A study reported that patients with BMS had a resting salivary flow of 0.40 (SD ± 0.27) mL/min and a stimulated salivary flow of 0.87 (SD ± 0.47) mL/min [3]. Overall research shows, that patients with BMS have a lowered non-stimulated salivary flow and a normal stimulated salivary flow and that a combined therapy shows a miniscule improvement of salivary flow, which in our case could be by the fact that patients after having multiple appointments felt calmer, since anxiety, depression and stress can affect salivary flow rates [2,5,30].

Overall there are not many studies that describe either using treatment options as vitamin B replacement therapy and zinc replacement therapy or topical capsaicin rinse therapy. This is the only study, that has looked into both.

By using a combined vitamin B and zinc treatment and the following treatment with capsaicin, it is possible to greatly reduce the number of patients with BMS and reduce the number of patients that might need a more serious drug treatment. Many parameters were checked before and after treatment. The limitation of the study is that there is no placebo group to compare the existing results and that not all the study participants consented on having all of their clinical data collected. Another limitation of the study is the fact, that the number of subjects is relatively small and that the administration of a combined treatment makes a direct interpretation of the results at the moment difficult.

## 5. Conclusions

In this study using a Vitamin B and zinc combined supplement therapy we were able to reduce pain/burning levels (in all three time frames of the day) for patients with BMS. For patients, that felt that the level of improvement was not satisfactory for their health, by receiving a topical capsaicin rinse were able to reduce their pain/ burning levels further. Vitamin B and zinc supplement therapy and topical capsaicin rinse therapy can be an effective way to decrease pain/burning sensation levels in patients with BMS. More research should be conducted to determine the benefit of either vitamin B and zinc supplement therapy or topical capsaicin rinse therapy, so that BMS patients have treatment options that have as few side-effects as possible.

## Figures and Tables

**Table 1:** Pain/Burning Levels Before and After Vitamin B and Zinc Supplement Therapy

No. of Patients	Time Period	Median + Interquartile Range(IQR) (Before Treatment)	Median + IQR (After Treatment)	Levels Decreased (Improvement)	Levels Did Not Change	Levels Increased (Worsening)	Wilcoxon (z-Score, *p* < 0.05)	% of Patients Noted Improvement
89	Morning	2 ± 3	1 ± 2	52	31	6	−5.503 (0.000)	58.43%
	Afternoon	4 ± 2	2 ± 2.5	61	23	5	−6.156 (0.000)	68.54 %
	Evening	5.5 ± 3	3 ± 3	60	23	6	−6.259 (0.000)	67.48%

**Table 2:** Pain/Burning Levels Before and After Oral Capsaicin Rinse Therapy

No. of Patients	Time Period	Median + IQR (Before Treatment)	Median + IQR (After Treatment)	Levels Decreased (Improvement)	Levels Did Not Change	Levels Increased (Worsening)	Wilcoxon (z, *p* < 0.05)	% of Patients Noted Improvement
20	Morning	2 ± 1	0 ± 2	9	10	1	−1.996 (0.046)	45%
	Afternoon	3 ± 1	1.5 ± 3	10	8	2	−2.613 (0.009)	50%
	Evening	4 ± 3	2 ± 6	10	8	2	−2.789 (0.005)	50%

**Table 3:** Salivary Flow Levels Before and After Treatment

Group	No. of Patients	Salivary Flow	Median + IQR (Before Treatment)	Median + IQR (After Treatment	Levels Increased	Levels Did Not Change	Levels Decreased	Wilcoxon (z, *p* < 0.05)	% of Patients Noted Improvement
Vitamin B and Zinc Supplement Therapy	48	Unstimulated	0.2 ± 0.19	0.21 ± 0.28	28	11	9	−2.908 (0.04)	58.33%
		Stimulated	0.8 ± 0.64	1 ± 0.53	31	8	9	−3.705 (0.000)	64.58%
Oral Capsaicin Rinse Therapy	10	Unstimulated	0.14 ± 0.21	0.225 ± 0.41	7	1	2	−1.955 (0.051)	70%
		Stimulated	0.94 ± 0.85	0.91 ± 0.61	7	1	2	−1.486 (0.137)	70%

## Data Availability

The data are not publicly available due to privacy concerns.

## Appendix A

**Table A1:** Pain/burning levels before and after Vitamin B and Zinc supplements.

No. of Patients	Time Period	Median + IQR (Before Treatment)	Median + IQR (After Treatment)	Levels Decreased (Improvement)	Levels Did Not Change	Levels Increased (Worsening)	Wilcoxon (z, *p* < 0.05)	% of Patients Noted Improvement
69 (Did not take part in the oral Capsaicin rinse therapy)	Morning	2 ± 3	1 ± 2	39	24	6	−4.589 (0.000)	56.5%
	Afternoon	4 ± 2	2 ± 2.8	47	19	3	−5.519 (0.000)	68%
	Evening	5 ± 2.5	3 ± 3.3	48	16	5	−5.658 (0.000)	69.5%
20 (Did take part in the oral Capsaicin rinse therapy)	Morning	2 ± 2	2 ± 1	13	7	0	−3.275 (0.001)	65%
	Afternoon	4 ± 1	3 ± 1	14	4	2	−2.635 (0.008)	70%
	Evening	6 ± 3	4 ± 3	12	7	1	−2.681 (0.007)	60%
